# Improved Numerical Calculation of the Single-Mode-No-Core-Single-Mode Fiber Structure Using the Fields Far from Cutoff Approximation

**DOI:** 10.3390/s17102240

**Published:** 2017-09-29

**Authors:** Wei Xu, Jia Shi, Xianchao Yang, Degang Xu, Feng Rong, Junfa Zhao, Jianquan Yao

**Affiliations:** 1 Institute of Laser and Optoelectronics, College of Precision Instrument and Optoelectronic Engineering, Tianjin University, Tianjin 300072, China; xuwei@tjpu.edu.cn (W.X.); tjushijia@tju.edu.cn (J.S.); yangxianchao@tju.edu.cn (X.Y.); xudegang@tju.edu.cn (D.X.); 2 Key Laboratory of Optoelectronic Information Science and Technology (Ministry of Education), Tianjin University, Tianjin 300072, China; 3 Tianjin Key Laboratory of Optoelectronic Detection Technology and Systems, Tianjin Polytechnic University, Tianjin 300387, China; rongfeng@tjpu.edu.cn (F.R.); zhaojunfa@tjpu.edu.cn (J.Z.)

**Keywords:** single-mode-no-core-single-mode fiber, guided-mode propagation analysis, numerical calculation, transmission spectra

## Abstract

Multimode interferometers based on the single-mode-no-core-single-mode fiber (SNCS) structure have been widely investigated as functional devices and sensors. However, the theoretical support for the sensing mechanism is still imperfect, especially for the cladding refractive index response. In this paper, a modified model of no-core fiber (NCF) based on far from cut-off approximation is proposed to investigate the spectrum characteristic and sensing mechanism of the SNCS structure. Guided-mode propagation analysis (MPA) is used to analyze the self-image effect and spectrum response to the cladding refractive index and temperature. Verified by experiments, the performance of the SNCS structure can be estimated specifically and easily by the proposed method.

## 1. Introduction

Recently, no-core fiber (NCF) has been widely investigated as a substitute for multimode fiber (MMF) etched by hydrofluoric acid (HF). Comparing NCF to MMF, the former has higher sensitivity to the external environment due to the lack of cladding. The single-mode-no-core-single mode fiber (SNCS) structure is reported as a tunable filter [1], an optical fiber sensor for liquid level [2], a refractometer [3,4], and a vibration fiber sensor [5], etc. SNCS structures are sensitive to the external refractive index; some use NCF directly [2,4] and others use MMF with HF corrosion [3,6]. Due to the high sensitivity of the refractive index (RI), the SNCS structure can be another platform for environmental sensing applications such as a modal interferometer made of photonic crystal fiber (PCF) [7], long period fiber grating (LPFG) fabricated in an endlessly single-mode photonic crystal fiber (ESM-PCF) [8], LPFG [9] or PCF [10] deposited by carbon-nanotube and a SPR fiber sensor [11], etc. With the increasing investigation of the SNCS structure coated by functional materials as a sensor [12,13,14], it is important to predict the refractive index (RI) response of the SNCS structure during the design phase. There are usually two methods to solve this problem: beam propagation method (BPM) and mode propagation analysis (MPA). A lot of works have been reported using BPM [15,16,17]. It will take a long time to calculate the transmission spectrum of the SNCS structure with centimeter NCF by BPM. This seriously affects the efficiency of sensor optimization for the SNCS structure. The transmission spectra of single mode-multi-mode-single mode fiber (SMS) structure are predicted by a one-way guided-mode propagation analysis (MPA) [18]. The MPA method is also used in the analysis of the high sensitivity refractometer [6] and the research of sensing characteristics [19] which are both based on the SMS. The operating principle of the SMS is multimode interference (MMI) excited between modes in the MMF section. The waveguide models based on slab waveguide and MMF have been used to investigate the self-imaging effect in MMIs [20,21,22]. The solution of mode propagation constants and mode coupling coefficients are two important steps in the MPA method. However, in most previous reports about MPA, the cladding refractive index (RI) of the MMF (without cladding etching) is not included [18]. It is suitable to employ the weakly guided approximation for MMF (without cladding etching). When it refers to NCF, if the weakly guided approximation is used to analyze the SNCS structure, the effect of the external RI on the calculated transmission spectra cannot be obtained. This is not in accordance with the experiment. It is well known that SNCS is sensitive to the external RI. Improved self-imaging for MMI involving the cladding RI for the first time is reported [23], but there is no explanation for why the cladding RI can be introduced in that way and the conclusion has not been tested experimentally.

In this paper, a modified model of NCF based on far from cut-off approximation, instead of weakly guided approximation, is proposed to investigate the spectrum characteristic and sensing mechanism of the SNCS structure. The MPA method is used to analyze the self-image effect of the SNCS structure and the spectrum response to cladding RI and temperature. The calculated spectral response agrees well with the experiment results. Using this method, the performance of the SNCS structure can be estimated precisely and efficiently. It is beneficial for optimizing fiber sensors which are made up of the SNCS structure coated by functional materials.

## 2. Theory Proposal

**Figure 1:**
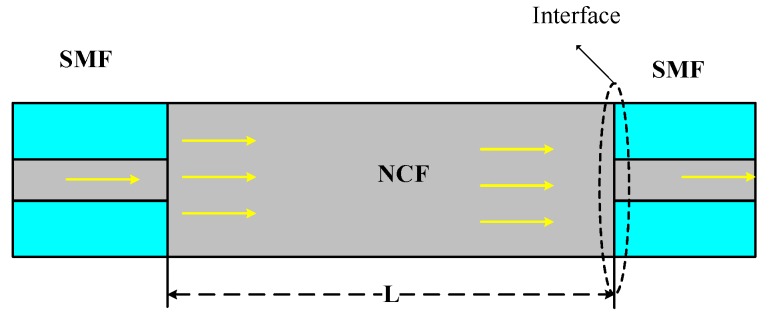
Schematic diagram of the single-mode-no-core-single mode fiber (SNCS) structure.

Figure 1 shows the schematic diagram of the SNCS structure which consists of a NCF and two standard SMFs. Assuming that the SMFs and NCF are aligned precisely, when the fundamental mode light in the SMF couples into the NCF, the high-order symmetric modes $LP_{0m}$ are excited.

The diameter of the NCF normally used is 125 μm, and the RI of the NCF and the external circumstance are not approximately equal, so the normalized frequency of the NCF is very large, which is defined as

(1) $$V=\frac{2\pi }{\lambda }a\sqrt{n_{1}^{2}-n_{2}^{2}}$$

where *a* is the radius of the NCF, λ is the wavelength, $n_{1}$ and $n_{2}$ represent the RI of the NCF and the external circumstance, respectively.

According to [24], the issue of the field transmission in the NCF belongs to the special case II—the fields far from cutoff. According to [25], the $LP_{0m}$ modes are equivalent to $HE_{1m}$ modes. We can obtain the normalized transverse wavenumbers of the $LP_{0m}$ modes in the NCF [24].

(2) $$u_{0m}\approx \mu_{0m}\left[1-\frac{n_{1}^{2}+n_{2}^{2}}{2n_{1}^{2}V}+\frac{1}{8}{\left(\frac{n_{1}^{2}+n_{2}^{2}}{2n_{1}^{2}V}\right)}^{2}\right]$$

(3) $$w_{0m}=\sqrt{V^{2}-u_{0m}^{2}}$$

where $\mu_{0m}$ is the *m*th order root of Bessel function $J_{0}$.

The power coupling efficiency represents the coupling of energy from the exciting field onto the different modes supported by the NCF. It is the power ratio of the high-order mode and the exciting field. The expression is [21]:

(4) $$\eta_{0m}=\frac{2{\left(\frac{\bar{\omega }}{a}\right)}^{2}exp\left[-{\left(\frac{\bar{\omega }}{a}\right)}^{2}\left(\frac{u_{0m}^{2}}{2}\right)\right]}{J_{0}^{2}\left(u_{0m}\right)+J_{1}^{2}\left(u_{0m}\right)+{\left(\frac{J_{0}\left(u_{0m}\right)}{K_{0}\left(w_{0m}\right)}\right)}^{2}\left(K_{1}^{2}\left(w_{0m}\right)-K_{0}^{2}\left(w_{0m}\right)\right)}$$

with $\bar{\omega }=\frac{a_{SMF}}{\sqrt{\ln2}}\left(0.65+1.619{V_{SMF}}^{-1.5}+2.879{V_{SMF}}^{-6}\right)$, where $a_{SMF}$ is the radius of the SMF, $V_{SMF}=\frac{2\pi }{\lambda }a_{SMF}\sqrt{n_{SMF\_core}^{2}-n_{SMF\_clad}^{2}}$.

Considering that the output and input SMF have the same parameters, the transmittance at the wavelength λ can be expressed by [18,21,22]

(5) $$T\left(\lambda \right)=10\log_{10}|\sum_{m}\eta_{0m}exp\left(j\beta_{0m}z\right){|}^{2}$$

where *z* is the length of the NCF, $\beta_{0m}$ is the propagation constant of $LP_{0m}$ mode which is related to $u_{0m}$ by the expression:

(6) $$u_{0m}=a\sqrt{k_{0}^{2}n_{1}^{2}-\beta_{0m}^{2}}$$

with

(7) $$k_{0}=\frac{2\pi }{\lambda }$$

Substituting Expression (6) into (2), the relationship between $\beta_{0m}$ and *V* can be concluded as

(8) $$\beta_{0m}=\sqrt{k_{0}^{2}n_{1}^{2}-\frac{\mu_{0m}^{2}}{a^{2}}{\left[1-\frac{n_{1}^{2}+n_{2}^{2}}{2n_{1}^{2}V}+\frac{1}{8}{\left(\frac{n_{1}^{2}+n_{2}^{2}}{2n_{1}^{2}V}\right)}^{2}\right]}^{2}}$$

The expression (2) is accurate to second order in $V^{-1}$ and the expression for $\beta_{0m}$ is accurate to first order in $u_{0m}/V$ [24].

Substituting Expressions (4) and (8) into (5), we can obtain the transmittance at different wavelengths. Through the wavelength λ scanning, the transmission spectrum of the SNCS structure can be obtained. Through the length *z* scanning, we can obtain the re-imaging distance which is defined as the propagation distance where the coupling efficiency is maximum.

In the above process, the RI characteristics of the SNCS structure can be analyzed by changing the external RI ($n_{2}$). In order to investigate the effects of temperature, we consider the influence of temperature on the RI and physical size of NCF, and assume that the external RI does not vary with temperature. The temperature dependence of the RI can be derived from [19] as

(9) $$n_{1}=n_{01}+\frac{dn_{1}}{dT}\left(T-T_{0}\right)$$

where $n_{01}$ is the RI of NCF at room temperature $T_{0}$.

The changes of length and overall radius induced by thermal expansion are taken as Δz=αzΔT and $\Delta a_{NCF}=\alpha a_{NCF}\Delta T$, respectively. α represents the thermal expansion coefficient and $\Delta T=T-T_{0}$.

## 3. Simulations and Experiments

As a numerical example, the SMF is assumed to be Corning SMF-28 with the following parameters: the RI of the core and cladding is 1.4681 and 1.4628, respectively, at 1550 nm and the radius of the core is 4.15 μm. The NCF (Prime Optical Fiber Co., Miaoli, Taiwan) is selected as the multimode fiber which is made of pure silica. Its dispersion can be obtained from the Sellmeier equation [26]. The mismatch of the RI between the SMF and NCF contributes to a reflection at the interface. The calculated reflectance with a simple Fresnel calculation is small (<0.8%), which is negligible in the calculations of the transmission spectrum. In the experiment, the NCFs are made of pure silica and the overall radii are 40 μm and 62.5 μm. The thermo-optic coefficient dn/dT and the thermal expansion coefficient α of fused silica is $1.06\times 10^{-5}{/}^{\circ }$C [27] and $5.0\times 10^{-7}{/}^{\circ }$C [28], respectively. The room temperature $T_{0}$ is set as $20{\;}^{\circ }$C. The SNCS structures are fabricated by a commercial fusion splicer (FSM-60s, Fujikura (Chinese) Co. Ltd., Beijing, China). The cleaving process and the fusion splicing procedure must be carefully controlled.

**Figure 2:**
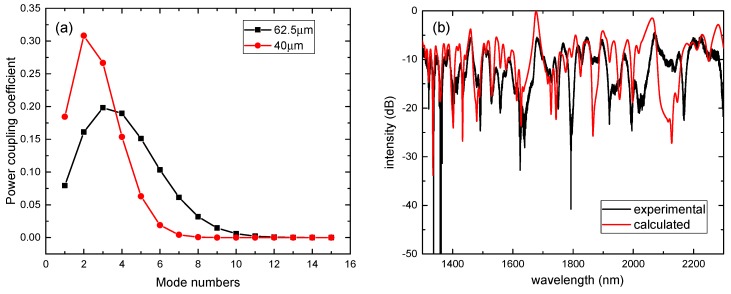
(**a**) The power coupling coefficient at different mode numbers with radius of 40 and 62.5 μm; (**b**) the calculative and experimental transmission spectra of SNCS structure with $a_{NCF}=62.5\;\mu$m and $z_{NCF}=54\;$ mm.

The algorithm is programmed in Matlab according to the description of Section 2. To determine the mode number, the power coupling coefficient is calculated . The power coupling coefficient, as a function of mode number for NCF with a radius of 40 and 62.5 μm, is shown in Figure 2a. When the mode number is more than 10, the power coupling coefficient is close to 0, which is similar to the reference [21]. In order to ensure the calculation accuracy, we selected the mode number m=15. The calculative and experimental transmission spectra are shown in Figure 2b in the case that the radius and length of NCF is 62.5 μm and 54 mm, respectively. The calculated results are in good agreement with the experimental results.

**Figure 3:**
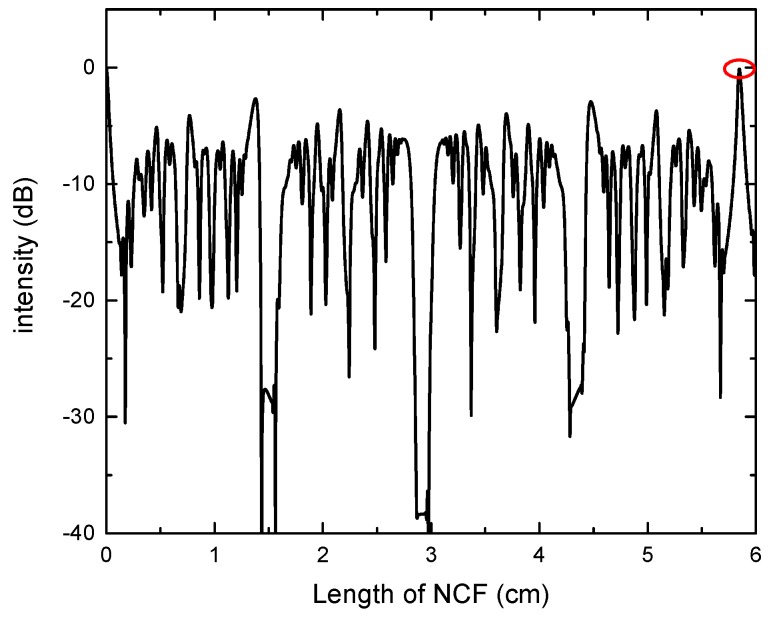
The power transmittance for different lengths of NCF (the minimum coupling loss is marked in the red circle).

**Figure 4:**
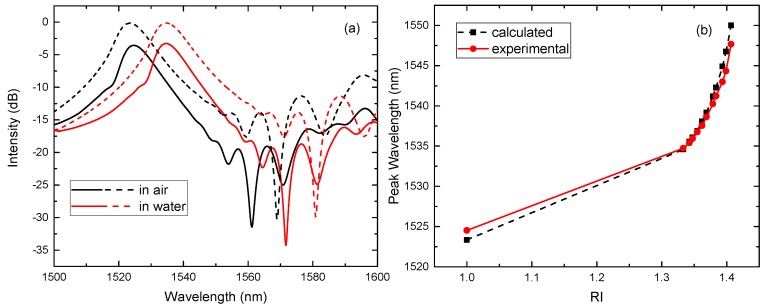
$a_{NCF}=62.5\;\mu$m and $z_{NCF}=59$ mm. (**a**) The calculated and experimental transmission spectra in air and water; (**b**) the calculated and experimental peak wavelength of the SNCS structure as the cladding RI increases.

As shown in Figure 3, the power transmittance for different lengths of NCF with radius of 62.5 μm is obtained through length scanning. The self-imaging distance for this numerical example is 58,471 μm with a coupling loss of −0.13 dB at the wavelength of 1550 nm. The self-imaging distance can be estimated in this way for different radii of NCF. The calculated and experimental transmission spectra in air and water are shown in Figure 4a. The length of the NCF is 59 mm. The solid line and the dotted line represent the result of the experiment and calculation, respectively. There are small discrepancies between the calculated and measured spectra. They may be caused by the manufacturing tolerances of the NCF, imperfect splicing between the SMF and NCF, and the insertion loss of the optical fiber connector, etc.

Under the condition of the parameters ($a_{NCF}=62.5\;\mu$m and $z_{NCF}=59$ mm), the calculated and experimental peak wavelength at different cladding (liquid) RIs is shown in Figure 4b. The peak wavelength increases monotonically in the calculation and experiment. The RI sensitivity at higher cladding RIs is bigger than that at lower cladding RIs in both cases. It is consistent with previous research [6]. The calculated sensitivity is 118 nm/RIU in the RI range from 1.333 to 1.3619 and 310 nm/RIU in the RI range from 1.3786 to 1.4067, the experimental sensitivity is 99 nm/RIU and 251 nm/RIU in the corresponding RI range.

**Figure 5:**
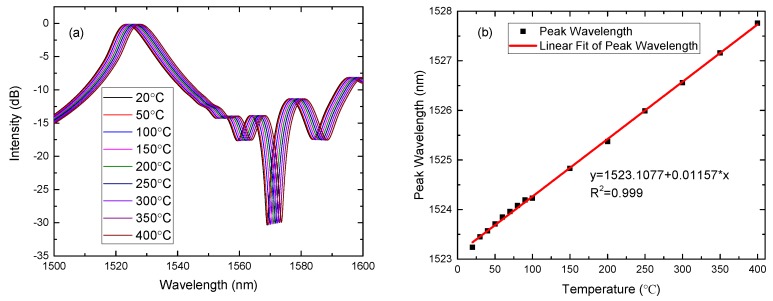
$a_{NCF}=62.5$ μm and $z_{NCF}=59$ mm. (**a**) The calculated transmission spectra as the temperature increases and (**b**) the response between the peak wavelength and temperature and the linear fitting.

Under the condition of the parameters ($a_{NCF}=62.5\;\mu$m and $z_{NCF}=59$ mm), the calculated transmission spectra at different temperatures are shown in Figure 5a. The temperature response of the peak wavelength and the linear fitting are shown in Figure 5b. The temperature sensitivity is about 12 pm/${}^{\circ }$C, in accordance with the experiment results of the reference [29].

As mentioned above, Expression (2) is under the condition of the fields far from cutoff. In order to discuss the applicability of Expression (2), the normalized frequency *V* at different radii and cladding RI are shown in Table 1 which is calculated at the wavelength of 1550 nm.

As shown in the Table 1, the *V* value is directly proportional to the radius of NCF and inversely proportional to the cladding RI. When the radius is 30 μm and the cladding RI is 1.44, the normalized frequency *V* is 16. In this case, the multimode transmission condition is still satisfied, but the number of the supported modes will be reduced. In practical applications, further reducing the radius of NCF will increase the difficulty of fabrication, and the thin NCF is fragile. The NCF does not satisfy the total internal reflection condition when the cladding RI is higher than 1.44, so the cladding RI must be less than 1.44. Under the two conditions above, Expression (2) is available, and the deduced MPA calculation method is feasible in predicting the transmission spectrum of the SNCS structure and calculating the self-image length.

**Figure 6:**
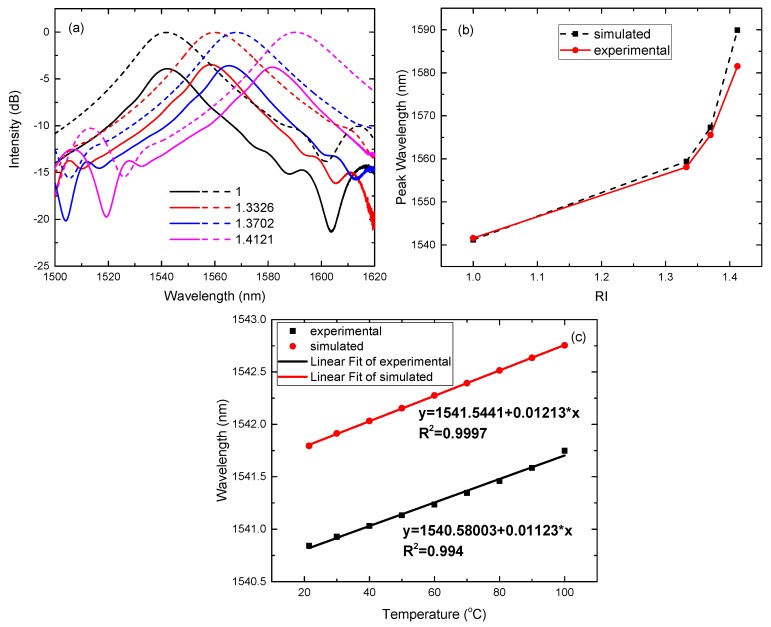
$a_{NCF}=40$ μm and $z_{NCF}=24\;\mathrm{mm}$. (**a**) The transmission spectra of the SNCS structure; (**b**) the response between the peak wavelength and cladding RI; (**c**) the response between the peak wavelength and temperature and the linear fit.

In order to verify the accuracy of the algorithm, we carried out a large number of experiments, and made comparisons with the calculation. Under the condition of the parameters ($a_{NCF}=40\;\mu$m and $z_{NCF}=24\;\mathrm{mm}$), the transmission spectra of the SNCS structure in different cladding RIs are shown in Figure 6a. The solid line is the experimental result and the dashed line is the calculated result. Figure 6b depicts the response between the peak wavelength and cladding RI in the simulated and experimental spectra. Figure 6c reveals the temperature response of the peak wavelength and the linear fit. There is a deviation of 1 nm in the peak wavelength between the experiment and calculation. We have to admit that it is difficult to control the length of NCF to be accurately 24 mm. The calculated sensitivity is 208 nm/RIU in the range from 1.3326 to 1.3702 and 540 nm/RIU in the RI range from 1.3702 to 1.4121. The experimental sensitivity is 198 nm/RIU and 381 nm/RIU in the corresponding RI range. By comparison, the sensitivity of RI increases as the diameter is reduced. The bandwidth of the bandpass filter spectral response in Figure 4 and Figure 5 increases as the diameter is reduced. This is beneficial for optimizing the fiber filter with the SNCS structure in the fiber laser.

**Figure 7:**
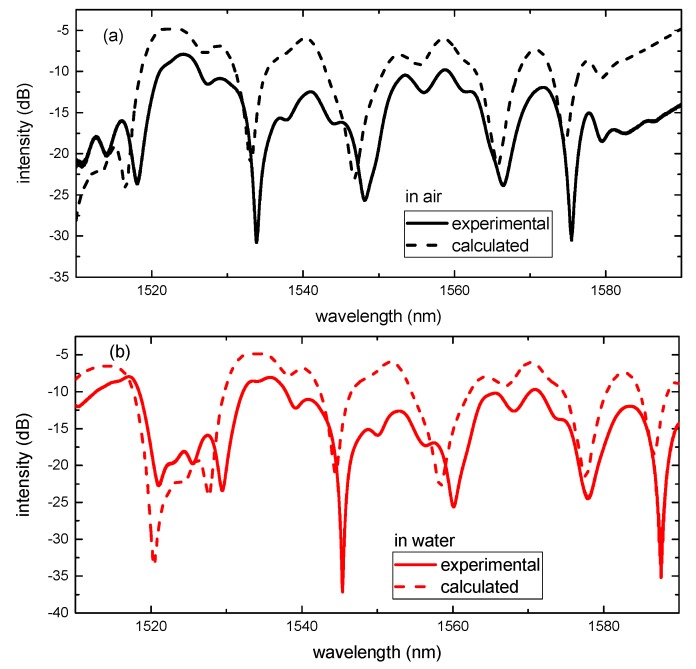
The simulated and experimental transmission spectra of the SNCS structure with $a_{NCF}=62.5\;\mu$m and $z_{NCF}=127\;\mathrm{mm}$ (**a**) in air; (**b**) in water.

The transmission spectra out of the bandpass filter are shown in Figure 7, in which the radius and the length of the NCF are 62.5 μm and 127 mm, respectively. The description of the solid line and dashed line is consistent with the preceding text. The experimental and simulation results are also in good agreement.

Through the algorithm, the transmission spectra, the RI and temperature response of the SNCS structure can be estimated accurately.

Employing similar methods to those in reference [23], the approximate formula of the self-image length can be obtained:

(10) $$z_{img}=10L_{\pi }=\frac{10\pi^{2}n_{1}a^{2}}{6.17\lambda (1-b+b^{2}/8)}$$

where $b=\frac{1}{2V}\left[1+{\left(\frac{n_{2}}{n_{1}}\right)}^{2}\right]$. The detailed derivation step of Expression (10) and the comparison of the self-imaging lengths obtained by an analytical approach and calculated by Expression (10) have been discussed, submitted as the supplementary material.

## 4. Conclusions

In this paper, an approximate expression of the normalized transverse propagation constant of no-core fiber is introduced according to optical waveguide theory. The transmission spectra of the SNCS structure are calculated by the MPA method and verified by experiments. The transmission spectra response to the cladding refractive index and temperature is also calculated. Employing this method, the performance of the SNCS structure can be estimated precisely and efficiently. It is beneficial for optimizing fiber sensors which are made up of the SNCS structure coated by functional materials. An approximate formula for the self-image length of the SNCS structure is derived, which provides theoretical support for the fabrication of SNCS structures.

## Figures and Tables

**Table 1:** The normalized frequency *V* at different radii and cladding RI.

	RI = 1	RI = 1.333	RI = 1.37	RI = 1.40	RI = 1.44
a = 62.5 μm	264.62	141.97	117.21	91.67	33.34
a = 52.5 μm	222.28	119.26	98.45	77.00	28.00
a = 45 μm	190.53	102.22	84.39	66.00	24.00
a = 40 μm	169.36	90.86	75.01	58.67	21.34
a = 30 μm	127.02	68.15	56.26	44.00	16.00
